# Maternal omega-3 fatty acids regulate offspring obesity through persistent modulation of gut microbiota

**DOI:** 10.1186/s40168-018-0476-6

**Published:** 2018-05-24

**Authors:** Ruairi C. Robertson, Kanakaraju Kaliannan, Conall R. Strain, R. Paul Ross, Catherine Stanton, Jing X. Kang

**Affiliations:** 1 Laboratory for Lipid Medicine and Technology, Department of Medicine, Massachusetts General Hospital and Harvard Medical School, Boston, MA USA; 2 Teagasc Moorepark Food Research Centre, Fermoy, Co. Cork Ireland; 3 APC Microbiome Ireland, University College Cork, Cork, Ireland

**Keywords:** Microbiome, Microbiota, n-3 PUFA, Obesity, Maternal diet

## Abstract

**Background:**

The early-life gut microbiota plays a critical role in host metabolism in later life. However, little is known about how the fatty acid profile of the maternal diet during gestation and lactation influences the development of the offspring gut microbiota and subsequent metabolic health outcomes.

**Results:**

Here, using a unique transgenic model, we report that maternal endogenous n-3 polyunsaturated fatty acid (PUFA) production during gestation or lactation significantly reduces weight gain and markers of metabolic disruption in male murine offspring fed a high-fat diet. However, maternal fatty acid status appeared to have no significant effect on weight gain in female offspring. The metabolic phenotypes in male offspring appeared to be mediated by comprehensive restructuring of gut microbiota composition. Reduced maternal n-3 PUFA exposure led to significantly depleted *Epsilonproteobacteria*, *Bacteroides*, and *Akkermansia* and higher relative abundance of *Clostridia*. Interestingly, offspring metabolism and microbiota composition were more profoundly influenced by the maternal fatty acid profile during lactation than in utero. Furthermore, the maternal fatty acid profile appeared to have a long-lasting effect on offspring microbiota composition and function that persisted into adulthood after life-long high-fat diet feeding.

**Conclusions:**

Our data provide novel evidence that weight gain and metabolic dysfunction in adulthood is mediated by maternal fatty acid status through long-lasting restructuring of the gut microbiota. These results have important implications for understanding the interaction between modern Western diets, metabolic health, and the intestinal microbiome.

**Electronic supplementary material:**

The online version of this article (10.1186/s40168-018-0476-6) contains supplementary material, which is available to authorized users.

## Background

The gut microbiota is a complex microbial ecosystem lining the intestinal tract that critically regulates host metabolism, immune responses and a number of other key physiological pathways [1–3]. The composition and function of the gut microbiota is profoundly influenced by environmental factors such as diet, mode of delivery at birth and antibiotic usage [4, 5]. Due to the essentiality of the commensal microbiota in host physiological homeostasis, environmental disturbance of gut microbiota composition and function can play a causal role in weight gain and metabolic dysfunction [3, 4, 6]. The microbiota is primarily established at birth through vertical transmission from the mother, while some reports also indicate microbial exposures in utero [7–9]. Indeed, the maternal vaginal microbiota closely resembles that of the infant microbiota soon after birth [7, 10]. Hence, disruption of the initial structuring of the microbiota in early-life may interfere with host metabolism and increase risk of later-life metabolic disease.

Disruption of the normal gut microbiota composition (“dysbiosis”) is often characterized by elevated relative abundance of pathogenic bacteria, such as lipopolysaccharide (LPS)-producing *Enterobacteriaceae*, or depletion of commensal species that maintain gut homeostasis, such as *Akkermansia muciniphilia* [11–13]. Recent research has examined how mode of delivery at birth, breast-feeding duration, and antibiotic use influence this structuring of the offspring microbiota [4, 14]. However, little is known about how the maternal prenatal or early-postnatal diet affects the phylogenetic architecture of the offspring microbiota and the subsequent effects this may have for metabolic disease risk in later life. Indeed, maternal nutritional inadequacies adversely affect fetal metabolic programming in the offspring leading to negative health consequences in later life [15]; however, the role of the microbiota in this trans-generational process remains underexplored.

Previous research has shown that a parental Western diet is associated with elevated plasma LPS and heightened colonic immune responses in standard chow-fed offspring [16]. These immune-modulating responses are dependent upon the comprehensive changes induced to the offspring microbiota, as a result of the parental diet. Indeed, maternal high-fat diets also induce compositional changes to the offspring microbiota in non-human primates [17]. Consequently, as the maternal microbiota correlates with that of the infant [7], optimization of maternal diet and microbiota composition may therefore enhance infant microbiota development.

Although it has been shown that high-fat diets contribute to perturbation in gut microbial balance and inflammation-induced obesity, recent evidence suggests that saturated and polyunsaturated fatty acids (PUFA) differ in their interaction with the gut microbiota and subsequent metabolic outcomes [18, 19]. Omega-3 (n-3) and omega-6 (n-6) PUFA play opposing roles in the inflammatory response [20]. n-6 PUFA typically upregulate inflammation by acting as precursors to pro-inflammatory eicosanoids, while n-3 PUFA resolve inflammation by competing within the same enzymatic pathway. The evolutionary ratio of n-6/n-3 PUFA has been estimated at 1:1; however, this has increased to 10–50:1 in the modern western diet which many have suggested has contributed to the epidemic of chronic inflammatory diseases such as obesity [21]. Indeed, there is convincing evidence that lowering the n-6/n-3 ratio can restore disrupted metabolism in the context of chronic disease [22, 23]. From an early-life perspective, lowering the n-6/n-3 ratio in obese mothers can reduce offspring weight gain and associated inflammatory outcomes in mice [24]. Similar results have been reported for improving insulin sensitivity [25]. Furthermore, observational and intervention studies in humans have found negative correlations for n-3 PUFA status and positive correlations for n-6 status and offspring adiposity [26, 27].

Indeed, n-6 and n-3 PUFA appear to have opposing effects on intestinal homeostasis. We have recently reported that, relative to dietary n-6 PUFA, n-3 PUFA reduce *Enterobacteriaceae* relative abundance, elevate relative *Bifidobacterium* abundance, and dampen intestinal inflammation partially through the upregulation of certain anti-microbial peptides in mice [28, 29]. Similarly, n-3 PUFA deficiency induces a state of gut dysbiosis through alteration of gut microbiota composition and impairment of short-chain fatty acid production [30]. Changes to gut microbiota composition induced by omega-3 deficiency in mice are also associated with behavioral impairments even before adolescence, suggesting an essential role for n-3 PUFA within the developing gut-brain axis [31].

Data on the role of maternal n-3 PUFA on offspring microbiota are limited. Some evidence suggests that high doses of maternal fish oil supplementation impair the immune response in offspring and lead to the overgrowth of pathogenic bacteria [32]. However, these studies utilized crude fish oil in pharmacologically excessive doses (18% energy), and hence, further studies are warranted to examine the relationship between nutritionally relevant maternal n-3 PUFA intake and offspring microbiota. In addition, the role of maternal n-3 PUFA intake in the context of offspring obesity and its association with gut microbiota has not been previously examined. Furthermore, there is a lack of evidence as to the differences between pre- and postnatal n-3 PUFA status for offspring metabolic health.

Based on previous evidence reporting the critical opposing roles that n-3 and n-6 PUFA play in microbiota-associated metabolic endotoxemia [28], we aim to investigate this in the context of maternal n-6/n-3 PUFA status and subsequent effects on obese offspring. To examine this, we utilize the *fat-1* transgenic mouse model, which is capable of endogenous conversion of n-6 PUFA to n-3 PUFA [33] and thereby eliminates confounding factors of diet. Therefore, using a single diet for both treatment groups, this model allows the generation of two phenotypes: (a) *fat-1* mice with a balanced tissue n-6/n-3 ratio (~ 1:1) and (b) wild-type (WT) mice with a high n-6/n-3 ratio similar to the Western diet (> 10:1). By examining the WT offspring of these two maternal genotypes and cross-fostering the offspring to mothers of different genotype at birth, we are able to identify the effect of maternal n-3 PUFA status during gestation and/or lactation on offspring health outcomes. Our results show that a lower maternal n-6/n-3 ratio during gestation and/or lactation significantly reduces weight gain and metabolic disruption in offspring fed a high-fat diet (HFD), which appears to be mediated through persistent restructuring of the gut microbiota. These studies provide novel evidence for the critical role of the maternal diet in early-life gut microbiota development and the subsequent impact on later-life metabolic disease states.

## Results

### Maternal fatty acid status during gestation and lactation differentially modulate offspring fatty acid profile

**Fig. 1:**
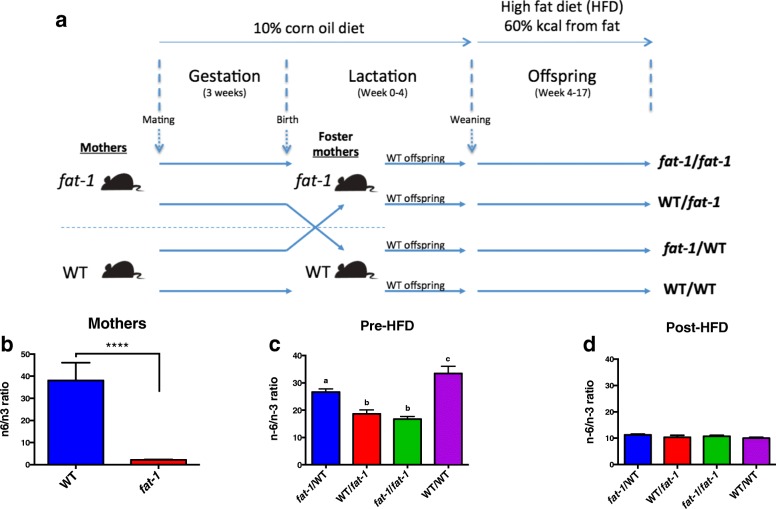
Experimental design and tail fatty acid profiles. **a** *fat-1* (*n* = 15) and WT mothers (*n* = 9) were mated while on a high-n-6 PUFA diet (10% corn oil). At birth, WT offspring were cross-fostered to different mothers for the period of lactation (4 weeks) to produce four experimental groups based on the mothers’ genotype (biological mother’s genotype/foster mother’s genotype): *fat-1*/WT (*n* = 8 males, *n* = 7 females), WT/*fat-1* (*n* = 9 males, *n* = 10 females), *fat-1*/*fat-1* (*n* = 9 males, *n* = 5 females), and WT/WT (*n* = 10 males, *n* = 10 females). During lactation, mothers were continued on a high-n-6 PUFA diet. After 4 weeks of lactation, offspring were weaned onto a high-fat diet (HFD) (60% kcal from fat) for 3 months during which body weights were assessed along with a number of other parameters. **b** WT mothers displayed a significantly greater tail n-6/n-3 ratio compared with *fat-1* mothers. **c** Following lactation for 4 weeks and prior to HFD, WT/WT male offspring had significantly greater tail n-6/n-3 ratio compared with all other groups. **d** However, after 3 months on a HFD, differences in tail n-6/n-3 fatty acid ratio were eliminated and there were no significant differences between groups. Data shown as mean ± SEM. *n* = 5–15 per group, *n* = 1–4 per cage. Bars with different letters are significantly different

To examine the effects of varying maternal fatty acid status during gestation and lactation on the tissue fatty acid profile of offspring, we created a cross-fostering protocol and measured offspring fatty acid composition at different time points (Fig. 1). Tissue fatty acid profiles of WT and transgenic *fat-1* mothers were assessed in addition to their WT offspring at weaning (4 weeks old) and following 3 months of HFD feeding (Fig. 1a). As expected, the n-6/n-3 PUFA ratio in tail tissue was significantly greater in WT mothers than in *fat-1* mothers (*p* < 0.0001, Fig. 1b). Maternal fatty acid profiles appeared to be transferred to offspring whereby, following 4 weeks of lactation, male offspring whom had a WT mother during both gestation and lactation (WT/WT) displayed a significantly greater n-6/n-3 ratio compared with all other groups (*p* < 0.0001, Fig. 1c). Conversely, offspring whom had a transgenic *fat-1* mother during both perinatal periods (*fat-1/fat-1)* displayed the lowest tail n-6/n-3 ratio. Interestingly, in the crossover groups, WT/*fat-1* offspring (those who had a WT mother during gestation and a *fat-1* mother during lactation) had a significantly lower n-6/n-3 ratio than *fat-1*/WT offspring, suggesting that the maternal fatty acid profile during lactation had a more pronounced effect on the offspring fatty acid profile than the maternal fatty acid profile during gestation.

Interestingly, differences in adolescent tail n-6/n-3 ratio as a result of maternal genotype were eliminated following 3 months of HFD feeding (Fig. 1d), and similarly, there were also no differences in n-6/n-3 PUFA ratio in offspring liver (Additional file 1: Table S3).

### Perinatal n-3 PUFA exposure reduces weight gain under high-fat diet

**Fig. 2:**
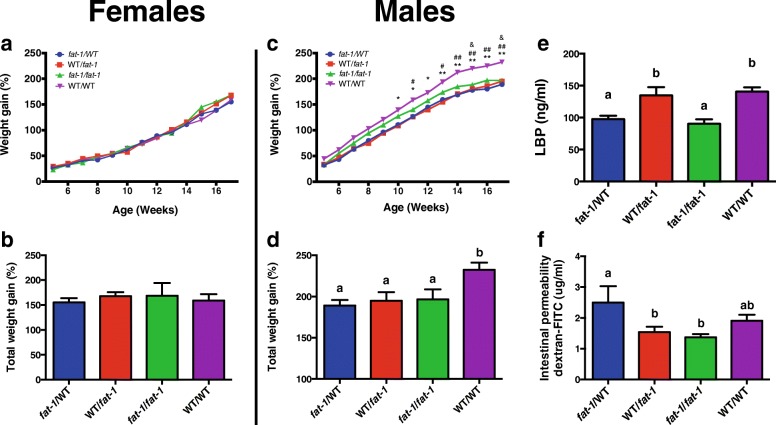
Weight gain and metabolic markers on a HFD. **a**, **b** There were no differences in weight gain between female groups. **c**, **d** WT/WT male offspring gained significantly more weight on a HFD than all other groups (repeated-measures two-way ANOVA (time and group) with Tukey’s post-hoc test). **e** *Fat-1/fat-1* displayed the lowest circulating LBP and intestinal permeability (**f**). Data shown as mean ± SEM. *fat-1*/WT: *n* = 8 males, *n* = 7 females; WT/*fat-1*: *n* = 9 males, *n* = 10 females; *fat-1*/*fat-1*: *n* = 9 males, *n* = 5 females; WT/WT: *n* = 10 males, *n* = 10 females. *n* = 1–4 animals per cage. Bars with different letters are significantly different. *p < 0.05 and **p < 0.01, WT/WT vs. WT/*fat-1*; #*p* < 0.05 and ##*p* < 0.01 WT/WT vs *fat-1*/WT; ^&^*p* < 0.05, WT/WT vs. *fat-1*/*fat-1*

To examine whether maternal fatty acid status affects offspring weight gain, offspring of WT and *fat-1* were fed a HFD (60% kcal from fat) for 3 months. There were no significant differences in offspring weights at week 4 prior to introduction of the HFD. In female offspring, there were no significant differences in weight gain between groups following 3 months HFD feeding (Fig. 2a, b). However, at 10 weeks of age and following 6 weeks of HFD feeding, WT/WT males had gained significantly more weight than WT/*fat-1* males (*p* < 0.05) suggesting that the influence of maternal n-3 PUFA status on offspring weight gain is gender-dependent. This effect of maternal fatty acid status on male offspring weight gain was strengthened with continuation of the HFD whereby WT/WT continued to gain significantly more weight than all other groups throughout the experimental period (Fig. 2c). At sacrifice, following 3 months of HFD, WT/WT mice had gained 232% body weight, which was significantly greater (*p* = 0.012) than all other groups (*fat-1*/WT—189%; WT/*fat-1*—195%; *fat-1*/*fat-1*—197%; Fig. 2d). Importantly, food intake did not differ significantly between groups. There were also no significant differences in body composition between groups (Additional file 1: Figure S1).

### Maternal n-3 PUFA dampen markers of metabolic disruption in offspring fed a high-fat diet

We then examined markers of metabolic disruption following HFD feeding to assess whether differences in offspring weight gain induced by maternal n-3 PUFA availability mediated metabolic health. Lipopolysaccharide-binding protein (LBP), a marker of metabolic endotoxemia, was lowest in *fat-1*/*fat-1* offspring (*p* < 0.05, Fig. 2e). Intestinal permeability (IP) is a measure of gut epithelial integrity relating to the passage of microbial endotoxins such as LPS into circulation. Again, the *fat-1*/*fat-1* group exhibited the lowest IP (*p* = 0.05, Fig. 2f)*.* However, at this time point, there were no observed differences between groups in concentrations of circulating serum cytokines IL-10, IL-1β, IL-6, MCP-1, or TNFα (Additional file 1: Figure S2A-E). Analysis of subcutaneous adipose tissue inflammation revealed no differences in mRNA expression of *TNFa*, *F4/80* or *CCL2*; however, *TLR4* expression was lower (*p* < 0.05) in *fat-1*/WT compared with both *fat-1*/*fat-1* and WT/WT (Additional file 1: Figure S2F-I).

There were subtle differences in glucose tolerance pre-HFD whereby WT/WT offspring had significantly greater circulating glucose (*p* < 0.05) at two time points during a glucose tolerance test (GTT). These differences were eliminated following HFD feeding (Additional file 1: Figure S3A-C). Insulin sensitivity also appeared to be differentially regulated post-HFD with the *fat-1*/*fat-1* group displaying the lowest insulin area under the curve (AUC) following glucose administration (*p* = 0.02, Additional file 1: Figure S3D-H).

### Maternal n-3 PUFA status has a profound influence on offspring microbiota composition in early life, which persists into adulthood

**Fig. 3:**
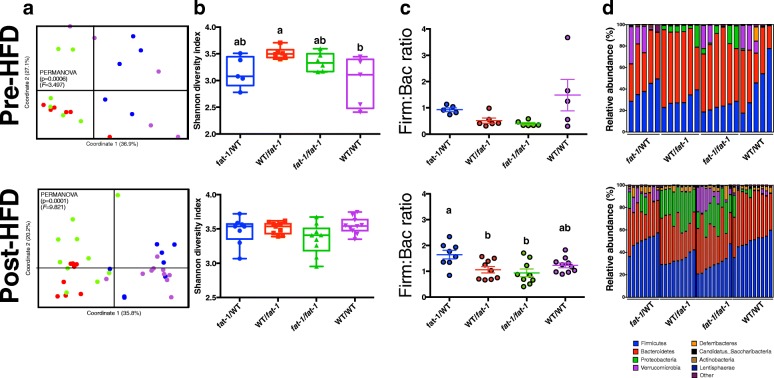
Phylum compositional and diversity differences in fecal microbiota between offspring of *fat-1* and WT mothers. **a** Analysis of beta diversity identified significant clustering of offspring groups by foster maternal genotype. **b** α-diversity as measured by the Shannon index did not differ between post-HFD groups, however, appeared to be reduced in WT/WT compared with WT/*fat-1* before HFD feeding. **c** The Firmicutes:Bacteroidetes ratio was lowest in *fat-1*/*fat-1* offspring. **d** Fecal microbiota composition differed significantly between offspring groups at phylum level and appeared dependent on foster mother genotype. Significant differences were determined by non-parametric analysis using the Kruskall-Wallis test followed by Mann-Whitney test. *n* = 5–10 per group, *n* = 1–4 per cage. Groups with different letters are significantly different

To examine whether differences in weight and metabolic health between offspring groups were mediated by changes in the gut microbiota, 16S rRNA gene sequencing was performed on fecal DNA of mothers and offspring both pre- and post-HFD feeding. 16S rRNA gene sequencing of the mothers’, offspring pre-HFD, and post-HFD fecal microbiota generated a total of 23 million sequenced reads. Principal coordinate analysis (PCoA) revealed distinct clustering of WT and *fat-1* mothers based on microbiota composition (Additional file 1: Figure S5A). Whole microbiome significance testing using PERMANOVA with Bray-Curtis dissimilarity index showed significant differences between *fat-1* and WT mothers (*p* = 0.0044, *F* = 4.874; Additional file 1: Figure S5A). In the offspring, clustering was evident among treatment groups, as assessed using PERMANOVA with Bray-Curtis dissimilarity index both pre-HFD (*p* = 0.0006; *F* = 3.497) and post-HFD (*p* = 0.0001, *F* = 9.821; Fig. 3a). Both prior to and after HFD feeding, offspring clustered significantly according to the foster mother’s genotype such that the *fat-1*/WT and WT/WT groups clustered together, and the *fat-1*/*fat-1* and WT/*fat-1* groups clustered together, according to Bray-Curtis dissimilarity index testing. Assessment of α-diversity using the Shannon Index revealed no significant differences between WT and *fat-1* mothers (Additional file 1: Figure S5B) or between post-HFD groups (Fig. 3b). However, microbial diversity as measured by the Shannon Index was significantly reduced in the WT/WT group compared with WT/*fat-1* prior to HFD feeding (Fig. 3b).

Relative abundances of phylum level taxa revealed distinct differences between groups (Fig. 3c, d). In mothers, Proteobacteria was the only phylum significantly different between groups following FDR correction (*p* = 0.05) and was present in greater relative abundance in *fat-1* females (Additional file 1: Figure S5C)*.* Taxonomic distribution appeared to be most significantly influenced by lactating/foster mother’s genotype as opposed to biological mother’s genotype. Hence, Proteobacteria was significantly greater in both WT/*fat-1* and *fat-*1/*fat-1* offspring both pre-HFD (*p* = 0.018) and post-HFD (*p* < 0.001). Verrucomicrobia were also highest in *fat-1*/*fat-1* offspring post-HFD (*p* = 0.023). Conversely, Firmicutes were significantly greater in *fat-*1/WT and WT/WT offspring post-HFD (*p* < 0.001).

**Fig. 4:**
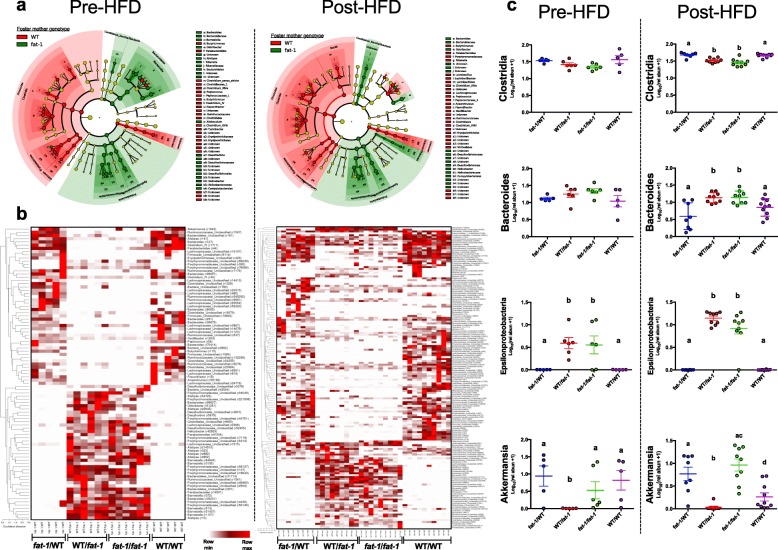
Genus level distribution of fecal microbiota between offspring of *fat-1* and WT mothers. **a** Least discriminant analysis of effect size (LEfSe) on clustered groups based on maternal genotype revealed that Firmicutes were more abundant in offspring of WT foster mothers whereas Bacteroidetes and Proteobacteria were more abundant in offspring of *fat-1* foster mothers. **b** Normalized data of fecal microbiota relative abundance revealed distinct differences between offspring groups. Microbiota composition appeared similar in groups of the same foster mother genotype. Data represents OTUs with significantly different relevant abundances between treatment groups (as determined by Kruskall-Wallis testing and Benjamani-Hocherg multiple correction testing). Each row represents an OTU labeled by lowest taxonomic description and OTU ID, normalized to the row maximum. Data normalized per taxonomic read. **c** Clostridia displayed significantly higher relative abundance in offspring of WT foster mothers. *Bacteroides* displayed higher relative abundance in offspring of *fat-1* foster mothers. *Akkermansia* displayed the highest relative abundance in *fat-1*/*fat-1* offspring. *Epsilonproteobacteria* were almost entirely depleted (< 0.1%) in offspring fostered to WT mothers. *n* = 5–10 per group, *n* = 1–4 per cage. Significant differences were determined by non-parametric analysis using the Kruskall-Wallis test followed by Mann-Whitney test and FDR correction by Benjamani-Hochburg testing. Groups with different letters are significantly different

Due to the significant PCoA clustering between groups based on foster mother’s genotype, offspring were grouped according to the foster mother’s genotype and linear discriminant analysis effect size (LEfSe) was performed on these two groups (1. WT/*fat-1* + *fat-*1/*fat-*1; 2. *fat-*1/WT + *fat-*1/*fat-*1) as a biomarker discovery tool to identify taxa that may be contributing to differences between groups (Fig. 4a and Additional file 1: Figure S4). LEfSe analysis elucidated the observed phylum level differences such that offspring whom had a WT foster mother were more abundant in species within the Firmicutes phylum (*Clostridia*) and offspring whom had a *fat-1* foster mother were more abundant in species of the Bacteroidetes (primarily *Bacteroides*) and Proteobacteria phyla (primarily *Epsilonproteobacteria*) (Additional file 1: Figure S5).

Further analysis at lower taxonomic levels revealed a number of taxa (13 genera pre-HFD and 26 genera post-HFD) that appeared to significantly differ between groups (Fig. 4b). *Clostridia* and *Parabacteroides* were significantly greater in *fat-1*/WT and WT/WT offspring post-HFD (Fig. 4c). Conversely, *Akkermansia* appeared to be significantly elevated in *fat-1*/*fat-1* offspring post-HFD. *Bacteroides* were also significantly lower in offspring with WT foster mothers. Interestingly, the reduced Proteobacteria observed in WT mothers and offspring fostered to WT mothers was driven primarily by depleted *Epsilonproteobacteria* (Fig. 4c) and *Deltaproteobacteria*, whereas relative abundance of *Gammaproteobacteria* was unchanged (Additional file 1: Figure S5). *Helicobacter*, a commensal member of the *Epsilonproteobacteria* order, which constituted 9–14% of the *fat-*1/*fat-1* and WT/*fat-1* post-HFD microbiota, was almost entirely depleted (< 0.1%) in offspring of WT foster mothers.

### Network interactions reveal host-microbiome interactions driven by fatty acid status

To assess the overall measure of correlation between the n-6/n-3 ratio-induced metabolic changes and microbiota, an RV coefficient was calculated. An RV coefficient of 0.456 (*p* = 0.001) was found between the relative abundance of microbial genera (pre- and post-HFD) and host phenotypes (mother and offspring pre-HFD n-6/n-3 ratio, LBP, IP, and total body weight gain).

**Fig. 5:**
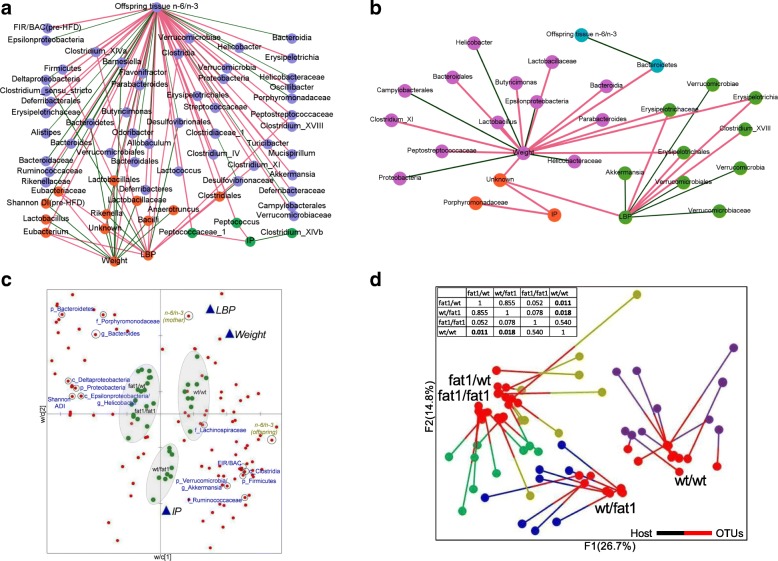
Network analyses of microbiome and host metabolic phenotype interactions. Host-microbiota interaction network built from Spearman’s non-parametric rank correlation coefficient (*P* < 0.05) between host parameters (mother and offspring pre-HFD n-6/n-3 ratio, body weight, IP, and LBP) and microbial parameters (pre- and post-HFD OTUs with FDR-corrected *p* values < 0.05, FIR/BAC ratio, and Shannon ADI) for **a** pre-HFD and **b** post-HFD. Each node was colored according to the modularity score and nodes were grouped as three (**a**) or four (**b**) modules. Lines represent statistically significant correlations and are colored red for positive and green for negative correlations. **c** Partial least square (PLS)-regression loading score plot illustrating the association between host parameters (dependent variables—*Y*) and microbial parameters (explanatory variables—*X*; red dots). Explanatory variables of interest with variable importance in the projection (VIP) scores 1 or > 1 were labeled with circles on the red dots. Samples from four different groups (*fat-1*/WT, WT/*fat-1*, *fat-1*/*fat-1*, WT/WT) were observed (green dots) and grouped using circles based on where they clustered on the plot. Leave-one-out cross-validation (LOO-CV) was applied. **d** Multiple factor analysis (MFA) using Spearman type principal component analysis of host and microbiota data. One end of the each connecting line for an observation indicates the host parameters (differently colored to indicate the groups) and another end (red) indicates the microbiota

Network-based analytical approaches have the potential to help disentangle complex host-microbe interactions [34]. Pairwise correlations between offspring n-6/n-3-induced changes in microbiota and host parameters with significant Spearman’s non-parametric rank correlation coefficient were employed to generate correlation networks for both pre-HFD and post-HFD (Fig. 5a, b). Correlation analysis with pre-HFD microbiota data resulted in a correlation network of 92 microbial parameters (taxa and α-diversity indices) and 4 host parameters (pre-HFD n-6/n-3 ratio, final body weight gain, LBP, and IP) for which at least one correlation could be found. The network consists of 118 edges (70 and 48 positive (green) and negative correlations (red) respectively) and 98 nodes (microbial and host parameters). Three modules were observed in the pre-HFD network (1. n-6/n-3 ratio; 2. weight + LBP; 3. IP). Accordingly, the largest module (n-6/n3 ratio) showed high modularity (blue nodes) comprised of both positive (P; red lines) and negative correlations (N; green lines) with a number of taxa, namely Firmicutes (P), Bacteroidetes (N), Proteobacteria (N), Clostridia (P), *Epsilonproteobacteria* (N), *Ruminococcaceae* (P), *Porphyromonadaceae* (N), *Bacteroides* (N), and *Akkermansia* (P) (Fig. 5a). A number of similar outcomes persisted in the post-HFD correlation network, whereby body weight was negatively associated with Proteobacteria and LBP negatively correlated with *Akkermansia* (Fig. 5b). Networks were also constructed between mother’s n-6/n-3 ratio, pre- and post-HFD microbiota data, and host parameters (Additional file 1: Figure S7).

To further investigate the identified groups of correlations between changes in microbiota composition and host parameters, we built partial least squares (PLS) models for selected correlations of interest created (Additional file 2; Goodness of fit—*Q*^2^_cum_ > 0.8). Combination of offspring pre-HFD n-6/n-3 ratios and all microbiota data explained 68, 72, and 61% variability in the body weight gain, LBP, and IP, respectively (Additional file 2: data models 4–6). A model created between all the host parameters (weight gain, LBP, and IP) and all microbiota data and mothers and offspring n-6/n-3 ratio (model 12) explained 45, 32, and 35% variability in the host parameters respectively (Fig. 5c). Variable importance in the projection (VIP) scores for the variables in each PLS model can be found in the Supplementary Data File 6 (M1–14).

Next, parameters contributing to the multivariate PLS models were compared with the corresponding identified modules in the correlation networks. Notably, 42 out of 50 pre-HFD microbial parameters, which exhibited direct correlations with offspring n-6/n-3 ratio in the network analysis (largest module), were presented with VIP values 1 or > 1 in the PLS model (model 8). The selected microbial parameters have been shown with *R*^2^ and VIP scores in the Supplementary data File 6 (M8). Twenty-eight out of 44 pre-HFD and 6 out of 9 post-HFD microbial parameters having correlations with mother’s n-6/n-3 ratio and LBP in the network analysis (largest module in the pre- and post-HFD networks) were presented with VIP values 1 or > 1 in the PLS model (model 3 and M3).

Finally, this was visualized using multiple factor analysis (MFA) that allowed microbial parameters to be superimposed onto the host parameters (Fig. 5d). Superimposed microbiome and host data were separated depending upon the n-6/n-3 ratio of both mother and foster mother. Pairwise comparison (Monte Carlo simulations with a *P* value = 0.008) between groups with superimposed host and microbiota data showed the importance of lactating mothers n-6/n-3 ratio when offspring were born from WT mothers.

## Discussion

In this study, we observed that the perinatal maternal tissue fatty acid profile profoundly and persistently restructures the offspring gut microbiota, which may have long-term implications for metabolic health. Maternal n-3 PUFA significantly reduced offspring weight gain into adulthood during high-fat feeding as has been demonstrated previously in humans and animals [26, 35, 36]. A number of mechanisms may contribute to this. Firstly, maternal tissue and milk n-3 PUFA correlate with umbilical cord and infant n-3 PUFA [37, 38]; therefore, the beneficial effects on weight may be attributed to direct nutrient transport from mother to infant and the subsequent anti-adipogenic effects of n-3 PUFA [39]. The intriguing aspect of these findings is that the *fat-1*/WT group had a higher n-6/n-3 ratio than the WT/*fat-1* group, suggesting that the offspring n-3 status is more influenced by the dietary n-3 PUFA during lactation, via milk, rather than maternal n-3 PUFA status during gestation. It has previously been reported that n-3/n-6 content in milk from *fat-1* females is greater than WT females [33]. Additionally, there appeared to be a strong gender difference between male and female offspring regarding weight gain as a result of maternal n-3 status. Maternal n-3 PUFA reduced weight gain in offspring males but not females. Such gender-dependent differences have been reported previously in humans, an effect which may be mediated by the interaction of female sex hormones with adipogenesis and fatty acid metabolism [40–42].

We also observed that maternal fatty acid status influenced immune regulation in the offspring, which may affect weight. It appeared that the lower n-6/n-3 ratio in *fat-1* mothers may have dampened maternal and placental inflammation, which induced an anti-inflammatory and anti-obesigenic environment in the offspring. This effect of maternal tissue fatty acid status on offspring inflammation has been demonstrated previously. Using the same *fat-1* model, it has been shown that maternal obesity induces maternal and placental inflammation, which is transmitted to the offspring resulting in a number of metabolic disruptions that are not evident in offspring of *fat-1* mothers [24]. Indeed, in this study, both IP and LBP were significantly reduced by maternal n-3 PUFA. This interesting finding suggests that the chronic low-grade inflammation that mediates the obesigenic phenotype [43] may be transferred from the mother to the infant during the perinatal period. This inflammatory phenotype appears to originate in the intestines through degradation of the intestinal barrier and hence translocation of bacterial endotoxins.

As has been reported previously, this inflammatory phenotype is induced by a disturbed microbiota [19, 44]. The composition of an infant’s microbiota is strongly influenced by that of the mother [7]. Therefore, the anti-inflammatory *fat-1* microbiota that has been described previously [28] may have been transmitted vertically to the offspring thereby reducing the microbiota-associated weight gain. The gut microbiota has a well-established role in energy metabolism and obesity by regulating energy harvest from macronutrients [6]. Interestingly, the observed differences in weight gain in this study were independent of dietary intake, which did not differ, suggesting that the WT/WT microbiota displayed an increased capacity for energy harvest. Previous hypotheses surrounding the “obesigenic” microbiota have concentrated on the energy-harvesting capacity of *Firmicutes* and the production of short-chain fatty acids (SCFA). Indeed, we observed this effect here whereby *Firmicutes* and their SCFA-producing families (*Lachnospiraceae* and *Ruminococcaceae*) were significantly greater in WT mothers and their foster offspring. We have previously demonstrated that differing ratios of dietary fatty acids significantly alter SCFA production in mice [30]. The role of the microbiota in regulating gut epithelial integrity and the subsequent inflammatory response has also been hypothesized to play a role in obesity [45, 46]. Here, we observed that IP and LBP were lowest in *fat-1/fat-1* offspring. These changes to IP were independent of changes to tight junction protein expression (Additional file 1: Figure S6). Conversely, *Akkermansia* was greater in offspring of *fat-1* mothers and hence may mediate the protective effect of maternal n-3 PUFA on weight gain in the offspring as has been shown previously in fish oil-fed mice [19]. There is growing evidence that *Akkermansia* plays a critical role in metabolic health and can reduce weight gain and metabolic endotoxemia in mice and humans [12, 13, 47]. *Epsilonproteobacteria*, and its genus *Helicobacter*, were also significantly depleted in offspring of WT foster mothers in response to a lack of perinatal n-3 PUFA. Our data extend on work of Ma et al. who reported that a maternal HFD in primates induced loss of non-pathogenic *Helicobacter* and *Campylobacter*, another member of *Epsilonproteobacteria*, in offspring [17]. Interestingly, offspring of non-human primates fed a high-fat diet exhibit reduced plasma n-3 PUFA [48].

This unique model and study design also allowed us to distinguish the role of the prenatal versus postnatal maternal tissue fatty acid profile on offspring outcomes. Interestingly, as with the n-6/n-3 ratio, the gut microbiota of the offspring appeared to strongly match that of the lactating/foster mother rather than the biological mother. Indeed, it has previously been shown in cross-fostering models that the nursing mother causes a permanent shift in the offspring microbiome [49]. It has been assumed that the fetus is sterile; however, recent studies may suggest otherwise [9]. The biological mother imprints a unique microbiota on the infant at birth [7]. The results reported here suggest that the labile “birth microbiota” is quickly and comprehensively altered by the foster mother, presumably through differences in milk/dietary fatty acids and the foster mother's microbiota following birth. These results would suggest that n-3 PUFA status in milk during lactation has a stronger impact on the infant microbiota than maternal fatty acid status during gestation. Hence, postnatal n-3 PUFA exposure may rescue and recover a “dysbiotic” offspring microbiota induced by the maternal prenatal n-3 PUFA insufficiency. Furthermore, despite the differences in n-3 and n-6 PUFA disappearing in adulthood after HFD, the observed differences in the microbiota persisted, suggesting that maternal fatty acid status and early neonatal feeding regime may have a persistent effect on the offspring microbiota throughout life.

## Conclusions

Much evidence exists indicating that obesity and its associated disorders have their origins in the fetal and neonatal periods. As the gut microbiota plays a critical role in the pathogenesis of these disorders and the chronic low-grade inflammation that defines them, nutrition research must now focus on maternal and early-life interventions that target the gut microbiota. This study has demonstrated that maternal fatty acid status persistently restructures the offspring microbiota and the associated metabolic homeostasis related to obesity. In addition, the unique transgenic model used here challenges the concept of a direct diet-microbiota interaction in obesity and instead uncovers the importance of the underlying tissue fatty acid profile in microbiota-metabolic interactions. These results have important implications for the current chronic disease epidemic. Excessive n-6/n-3 ratios in the Western diet have contributed to a trans-generational epidemic of chronic metabolic disease, which may be partially attributed to persistent gut microbiota dysbiosis. Consequently, maternal n-3 PUFA intake, especially during lactation, poses potential as an effective therapeutic measure to restore gut microbiota homeostasis and metabolic disturbances associated with modern chronic disease.

## Methods

### Animals and diets

Generation of transgenic *fat-1* mice was performed as previously described [33] followed by backcrossing onto a C57BL/6 background. *Fat-1* phenotype was confirmed by gas chromatography flame-ionization detection (GC-FID) following identification of increased tissue n-3 PUFA compared with WT. *Fat-1* genotype was confirmed by RT-PCR. Mice were housed in the Massachusetts General Hospital (MGH) animal facility in a biosafety room (level 2) in hard top cages with filtered air. Mice were maintained in a temperature-controlled room (22–24 °C) with a 12-h light/dark diurnal cycle. Food and water were provided ad libitum. A subset of 3-month-old female C57BL/6 WT mice was purchased from Charles River Laboratories and allowed to acclimatize to the facility conditions for 1 week prior to mating. *Fat-1* and WT mating pairs were fed a diet high in n-6 PUFA (AIN-76A with 10% corn oil) from LabDiet in order to maintain *fat-1* and WT phenotypes. At postnatal day (PND) 28, male and female offspring were weaned onto a high-fat diet (HFD) with 60% kcal from fat (D12492, Research Diets Inc.). Detailed fatty acid profiles of both diets are outlined in Additional file 1: Table S1. Body weight and food intake were measured weekly using an electrical balance. Body composition (fat mass, lean mass, water mass) was assessed on the day of sacrifice using a Minispec mq bench-top NMR spectrometer (Bruker Instruments). Animals were sacrificed using CO_2_. Dissected tissues were flash frozen in liquid nitrogen. All animal procedures in this study were performed in accordance with the guidelines approved by the MGH Subcommittee on Research Animal Care.

### Breeding and cross-fostering

Three-month-old female *fat-1* (*n* = 15) and WT (*n* = 9) mice were mated with age-matched WT males. Mating pairs were housed in individual cages, and males were separated from the females following confirmation of pregnancy. Within 48-h of parturition, newborn litters were fostered to new mothers until weaning, at PND 28. Briefly, the newborn litter was removed from the biological mother’s cage then mixed with the bedding of the foster mother in the hand of the investigator. The litter was then placed in the empty nest of the new foster mother. The foster mother was held above the new litter until she urinated on the litter in order to disguise their scent. The foster mother pairs were chosen such that offspring were fostered to mothers whom had given birth within 48 h to a litter of similar size. The cross-fostering procedure was carried out in order to generate offspring of four distinct experimental groups as follows: *n6-n3 group*—WT biological mother, cross-fostered to *fat-1* mother; *n3-n6 group*—*fat-1* biological mother, cross-fostered to WT mother; *n3-n3 group*—*fat-1* biological mother, cross-fostered to new *fat-1* mother; *n6-n6 group* – WT biological mother, cross-fostered to new WT mother. Cross-fostering was carried out in the *n3-n3* and *n6-n6* groups as a control to the cross-fostering procedure in the other two groups. The study design is outlined in Fig. 1.

At PND 10, the tails of the offspring were clipped with a scissors and genotyping was performed on the tail tissue by RT-PCR. Following confirmation of genotype, *fat-1* mice were removed from the litter such that only the WT offspring remained. At PND28, WT offspring were separated from their mothers, grouped in separate cages (randomized by experimental group, *n* = 1–4 animals per cage, *n* = 3–4 cages per experimental group) and weaned onto the HFD.

### Fatty acid analysis

Fatty acid analysis of tail and liver tissues was performed as previously described [50]. Briefly, frozen tissue samples were ground to a powder under liquid nitrogen using a mortar and pestle. Lipid extraction and fatty acid methylation was performed by the addition of 14% (*w*/*v*) boron trifluoride (BF3)-methanol reagent (Sigma-Aldrich) followed by heading at 100 °C for 1 h. Fatty acid methyl esters (FAME) were analyzed using a fully automated HP5890 gas chromatography system equipped with a flame-ionization detector (Agilent Technologies, Palo Alto, CA). The fatty acid peaks were identified by comparing their relative retention times with the commercial mixed standards (NuChek Prep, Elysian, MN), and area percentage for all resolved peaks was analyzed by using a PerkinElmer M1 integrator.

### Intestinal permeability

Intestinal permeability was performed as described previously [28]. Briefly, mice were fasted for 6 h and then FITC-dextran (70kDA, Sigma-Aldrich, in PBS solution) was administered to mice by oral gavage at a dose of 600 mg/kg body weight. Following gavage, blood samples were collected from the facial vein after 90 min. Serum was diluted with an equal volume of PBS, and fluorescence intensity was measured using a fluorospectrophotometer (PerkinElmer) with an excitation wavelength of 480 nm and an emission wavelength of 520 nm. Serum FITC-dextran concentration was calculated from a standard curve generated by serial dilution of FITC-dextran in PBS.

### Serum LBP

Concentrations of lipopolysaccharide-binding protein (LBP) in serum were assayed using a commercial ELISA kit (NeoBioLab, Cambridge, MA) according to the manufacturer’s instructions.

### Stool DNA extraction and 16S rRNA gene sequencing

Bacterial genomic DNA was extracted from mice fecal pellets using the QIAmp DNA Stool Mini Kit (Qiagen, UK) according to the manufacturer’s instructions. DNA was quantified and purification was subsequently assessed by measuring absorbance and determining the A260/A280 ratio. DNA was stored at − 20 °C until analysis.

16S rRNA gene sequencing library preparation was performed on DNA samples according to the Illumina 16S rRNA gene sequencing library protocol in order to generate V3-V4 amplicons. DNA samples were subjected to an initial PCR reaction utilizing primers specific for amplification of the V3-V4 region of the 16S rRNA gene (Forward primer 5′ TCGTCGGCAGCGTCAGATGTGTATAAGAGACAGCCTACGGGNGGCWGCAG; reverse primer 5′ GTCTCGTGGGCTCGGAGATGTGTATAAGAGACAGGACTACHVGGGTATCTAATCC). Clean-up and purification of the PCR product was performed using the Agencourt AMPure XP system (Labplan, Dublin, Ireland). Following clean-up and purification, a second PCR reaction was performed in order to incorporate a unique indexing primer pair to each sample (Illumina Nextera XT indexing primers, Illumina, Sweden). The PCR products were purified a second time using the Agencourt AMPure XP system. Quantification of samples was performed using the Qubit broad range DNA quantification assay kit (Bio-Sciences, Dublin, Ireland). Following quantification, samples were pooled in equimolar amounts (10 nM) and sequenced at Clinical-Microbiomics (Copenhagen, Denmark) using Illumina MiSeq 2 × 300 bp paired-end sequencing.

### 16S rRNA gene sequencing bioinformatics analysis

The 64-bit version of USEARCH 8.1.1825 [51] and mother v.1.36.1 [52] were used for bioinformatic analysis of the sequence data. These were used in combination with customized in-house programs, the process of which is detailed precisely below.

Following tag identification and trimming (performed natively in the sequencing machine), all sequences from all samples were pooled using a perl script (bash command "cat", e.g. "cat *.R1.fastq > pool.R1.fastq"). Using USEARCH’s—fastq_mergepairs command, paired-end reads were merged, truncating reads at a quality score of 4, requiring at least 100 bp overlap and a merged read length between 300 and 600 bp in length and less than one expected errors in the merged read ("usearch8.1.1825_i86osx64 -fastq_mergepairs pool.R1.fastq -reverse pool.R2.fastq -fastq_minmergelen 300 -fastq_maxmergelen 600 -fastq_minovlen 100 -fastq_merge_maxee 1 -fastaout merged.fasta -relabel -"). Using mothur's trim.seqs command, sequences with ambiguous bases, without perfect match to the primers, or homopolymer length greater than 8 were discarded and primer sequences trimmed. Using USEARCH’s—derep_fulllength command, sequences were then strictly dereplicated discarding clusters smaller than 5.

Using USEARCH’s—cluster_otus command, sequences were clustered at 97% sequence similarity, using the most abundant strictly dereplicated reads as centroids (" usearch8.1.1825_i86osx64 -cluster_otus rua16s.sorted.fasta -otus rua16s.clustered.fasta --uparseout rua16s.clustered.table"). Using USEARCH’s—uchime_ref [53] command, suspected chimeras were discarded based on comparison with the Ribosomal Database Project classifier training set v9 [54]. Using mothur’s classify.seqs command, taxonomic assignment of OTUs was performed using the method by Wang et al. [55] with mothur’s PDS version of the RDP training database v14 ("classify.seqs(fasta=abrecovery.fasta, template=otu.fasta, taxonomy=trainset14_032015.pds.fasta, method=wang)"). The Wang parameters used were ksize = 8, iters = 100, and cutoff = 0. A bootstrap threshold of 80 was subsequently applied using *R* and values < 80 were assigned as “unclassified”. Alpha diversity was calculated using mothur’s Shannon command. To reduce bias from variation in sample read numbers, samples were rarefied to the sample with the lowest read count, 10,597 reads. Rarefaction can introduce bias into data and thereby affect outcomes [56, 57]. To ensure our data processing did not affect data outcomes, we performed PERMANOVA with Bray-Curtis dissimilarity testing to examine whether rarifying caused differences to the sequencing data. No significant differences were observed between rarified and non-rarified data. Similarly no significant differences were observed in alpha diversity (Shannon index) or between any single OTU (Kruskall-Wallis followed by Mann-Whitney testing) between rarified and non-rarified data.

### Statistical analysis

Statistical analysis was performed using SPSS (v19, NY, USA), GraphPad Prism (v6, CA, USA) and R (v3.2.4). One-way analysis of variance (ANOVA) was performed to assess differences between groups followed by Tukey’s or LSD post-hoc test. Repeated-measures two-way ANOVA (time and group) with Tukey’s post-hoc test was used for weight gain data. For 16S rRNA gene sequencing data, principal coordinate analysis was conducted using PAST Software (v3.18) with Bray-Curtis dissimilarity testing. To assess whether significant differences existed between specific taxa, non-parametric analysis was performed using the Kruskall-Wallis test followed by Mann-Whitney test. False discovery rate (FDR) analysis was subsequently performed using the Benjamani-Hochburg method and significance was calculated at *q* < 0.05.

Linear discriminant analysis (LDA) effect size (LEfSe) is a biomarker discovery tool for high-dimensional data that provides effect size estimation [58]. Microbiota-based biomarker analysis was performed with LEfSe using the online galaxy server (https://huttenhower.sph.harvard.edu/galaxy/). LDA scores (> 3.0) derived from LEfSe analysis were used to show the relationship between taxon using a cladogram (circular hierarchical tree). Levels of the cladogram represent, from the inner to outer rings, phylum, class, order, family, and genus.

For host-microbiome interaction analysis, an RV coefficient (a multivariate generalization of the Pearson correlation coefficient) was calculated between the microbial (pre- and post-HFD) and host parameters (mother and offspring pre-HFD n-6/n-3 ratio, LBP, IP, and body weight). The data was rescaled from 0 to 1 before analysis.

Pairwise correlations between each taxon and host parameter were calculated using the Spearman’s non-parametric rank correlation coefficient. Network-based analytical approaches have been used previously to disentangle host-microbe interactions [59, 60]. Data was rescaled from 0 to 1 before analysis. Based on these correlation coefficients, a correlation network (label adjust and no overlap layout) was built where nodes represent either a taxon or a host parameter. For each taxon and host parameter, an undirected edge was added between the corresponding nodes in the correlation network. Edges (red links indicate positive and dark green links indicate negative associations) represent statistically significant correlations (*P* < 0.05). Correlations were calculated in PAST software version 2.17 and the network was visualized in Gephi Graph Visualization and Manipulation software version 0.9.2. Nodes were colored based on a “modularity” community detection algorithm. A “module” in the network is a set of nodes connected to each other by many links, while connected by few links to nodes of other groups, so modules are elementary units of any biological network (each assigned a unique color). Degree centrality of nodes was employed as an index of node centrality.

Partial least squares regression (PLS-R) was used to associate the microbial composition with host parameters including jackknife-based variable selection as reported previously [60]. For all models, the data were rescaled from 0 to 1 before PLS-R and centered as well as reduced during PLS-R. Leave-one-out cross-validation (LOO-CV) was applied. The *Q*^2^ cumulated index (*Q*^2^_cum_) was used as a measure of the global goodness of fit, the predictive quality of the models and to test the validity of the model against over-fitting. A *Q*^2^_cum_ threshold of > 0.8 was applied. The resulting plot displays the dependent variables on the c vectors and the explanatory variables on the w* vectors which allows visualizing the global relationship between the variables. The w* are related to the weights of the variables in the models. The results are also presented in PLS scatter plots for subject clustering and variables. The *R*^2^ (coefficient of determination) indicates the percentage of variability of the dependent variable (*Y*) which is explained by the explanatory variables (*X*). The relative importance of each *x*-variable is expressed by variable importance in the projection (VIP) values. VIP-value 1 or > 1.0 is considered influential and > 1.5 as highly influential. Results of all of the above mentioned statistics were given in the Supplementary file 6. All analyses were performed using precise algorithm in the XLSTAT software version 2017.6.

Associations between the host and microbiome data sets were also assessed by multiple factor analysis (MFA). The methodology of the MFA breaks up into two phases: (i) a principal component analysis (PCA) (Spearman type) successively carried out for each dataset (Dataset 1, host parameters; Dataset 2, pre- and post-HFD microbiota data) which stores the value of the first eigenvalue of each analysis to then weigh the various datasets in the second part of the analysis. (ii) A weighted PCA on the columns of all the datasets leads to each indicator variable having a weight that is a function of the frequency of the corresponding category. After these two phases, the coordinates of the projected points in the space resulting from the MFA are displayed. The projected points correspond to projections of the observations in the spaces reduced to the dimensions of each dataset. Based on the eigenvalues of the weighted PCA, the first two factors (F1/F2) almost covered 60% of the variability in this analysis. To test whether the four groups with superimposed host and microbiome data were separated from each other, Kruskal-Wallis testing was performed on the coordinates of the projected points and *p* values were obtained using 10,000 Monte Carlo permutations. One end of each line for an observation indicates the host parameters (differently colored to indicate the groups) and another end (red) indicates the microbiota.

## Additional file

Additional file 1: **Figure S1.** Male and female body composition. **Figure S2.** Serum cytokines and subcutaneous adipose tissue inflammatory gene expression did. **Figure S3.** Glucose tolerance and insulin tolerance testing. **Figure S4.** LDA scores following LEfSe analysis of pre-HFD and post-HFD microbiota grouped by foster mother genotype. **Figure S5.** Mothers microbiota and offspring proteobacteria abundance. **Figure S6.** Ileal tight junction protein expression. **Figure S7.** Correlation network of maternal fatty acid status and offspring microbiota. **Table S1.** Fatty acid profile of diet. **Table S2.** Tail fatty acid profiles of mothers and offspring before and after high-fat diet feeding. **Table S3.** Liver fatty acid profiles of offspring after high-fat diet feeding. **Table S4.** Primer sequences for qPCR. (DOCX 5230 kb)

Additional file 2: Supplementary Data. (XLSX 75.6 kb)
