# Supplementary material for: Maternal omega-3 fatty acids regulate offspring obesity through persistent modulation of gut microbiota
Source: Microbiome. 2018 May 24;6:95. doi: 10.1186/s40168-018-0476-6 (PMC5968592; doi:10.1186/s40168-018-0476-6)
Supplement: Supplementary file 1 — Figure S1. Male and female body composition. Figure S2. Serum cytokines and subcutaneous adipose tissue inflammatory gene expression did. Figure S3. Glucose tolerance and insulin tolerance testing. Figure S4. LDA scores following LEfSe analysis of pre-HFD and post-HFD microbiota grouped by foster mother genotype. Figure S5. Mothers microbiota and offspring proteobacteria abundance. Figure S6. Ileal tight junction protein expression. Figure S7. Correlation network of maternal fatty acid status and offspring microbiota. Table S1. Fatty acid profile of diet. Table S2. Tail fatty acid profiles of mothers and offspring before and after high-fat diet feeding. Table S3. Liver fatty acid profiles of offspring after high-fat diet feeding. Table S4. Primer sequences for qPCR. (DOCX 5230 kb) [file 40168_2018_476_MOESM1_ESM.docx]

**Additional file 1**

**Figure S1. Male and female body composition.** There were no differences in subcutaneous fat weight between offspring male groups (p=0.09). There were no differences between female groups in body composition. Data shown as mean ± SEM. n= 8-10 per group.

**Figure S2. Serum cytokines and subcutaneous adipose tissue inflammatory gene expression did. (a-e)** There were no significant differences between offspring groups in circulating serum cytokines. **(f-i)** There were no significant differences in subcutaneous adipose tissue expression of CCL2, F4/80 or TNFα. TLR4 expression was significantly lower in *fat-1*/WT. Data shown as mean ± SEM. n= 8-10 per group.

**Figure S3. Glucose tolerance and insulin tolerance testing. (a)** Maternal n-3 PUFA status did not significantly impact fasting glucose either prior to or after 10 weeks HFD feeding. **(b)** WT/WT glucose was significantly higher than WT/*fat-1* at two time points during the pre-HFD GTT however no significant differences were observed post-HFD. **(c)** No significant differences were observed in the AUC of the GTT either prior to or after HFD feeding. **(d-e)** During the insulin tolerance test (ITT) at week 12, no significant differences were observed in baseline fasting glucose or insulin. **(f-h)** The *fat-1fat-1* group had the lowest glucose at three time-points during the ITT, which led to a significantly lower AUC. Homeostatic model assessment of insulin resistance (HOMA-IR) score was not significantly affected. Data shown as mean ± SEM. n= 7-12 per group. *p<0.05 **p<0.01, WT/WT vs. WT/*fat-1,* #p<0.05 ##p<0.01, *fat-1*/WT vs. WT/WT.

**Figure S4. LDA scores following LEfSe analysis of pre-HFD and post-HFD microbiota grouped by foster mother genotype.** LEfSe identified a number of members of Bacteroidetes (*Bacteroides*) and Proteobacteria (*Epsilonproteobacteria and Deltaproteobacteria*) phyla which displayed the highest LDA scores in offspring fostered to a *fat-1* mothers and members of Firmicute*s* (*Clostridia*) which had the highest scores in offspring fostered to WT mothers.

**Figure S5. Mothers microbiota and offspring proteobacteria abundance**. **(a-b)** Whole microbiome significance testing using PERMANOVA with Bray-Curtis similarity index showed significant differences between *fat-1* and WT mothers but no differences were observed in alpha diversity. **(c)** Phylum level distribution varied widely between WT and *fat-1* mothers. **(d)** PCA of top 10 most abundant taxa at family level in mothers. **(e-l)** The elevated *Proteobacteria* abundance offspring of *fat-1* mothers and their foster offspring was driven by *Deltaproteobacteria* and *Epsilonproteobacteria*, particularly *Helicobacter*, whereas no differences were observed in *Gammaproteobacteria*. n= 7-12 per group. Groups with different letters are significantly different. *p<0.05, **p<0.01.

**Figure S6. Ileal tight junction protein expression.** WT/WT trended towards lower occluding expression (p=0.08) and had significantly greater claudin expression than *fat-1*/WT. n=8-10 per group. Data shown as mean ± SEM.

**Figure S7. Correlation network of maternal fatty acid status and offspring microbiota.** Host-microbiota interaction network built from Spearman's nonparametric rank correlation coefficient (*P* < 0.05) between maternal n-6/n-3 ratio and offspring microbial parameters (pre and post HFD OTUs with FDR corrected p-values <0.05, FIR/BAC ratio and Shannon ADI) for **(a)** pre-HFD and **(b)** post-HFD.

**Table S1.** Fatty acid profile of diet

| **Fatty acid** | **Diet** | |
| --- | --- | --- |
|  | **10% corn oil** | **HFD** |
| **C8:0** | 0.11 | 0 |
| **C10:0** | 0.27 | 0.04 |
| **C12:0** | 0.7 | 0.08 |
| **C14:0** | 0 | 1.1 |
| **C15** | 0 | 0.08 |
| **C16** | 0 | 19.6 |
| **C16:1** | 0.5 | 1.34 |
| **C17:0** | 0 | 0.35 |
| **C18:0** | 2.36 | 10.6 |
| **C18:1** | 28.2 | 34.0 |
| **C18:2 n-6** | 52.3 | 28.7 |
| **C18:3 n-3** | 1.1 | 2.04 |
| **C20:0** | 0 | 0.16 |
| **C20:1** | 0 | 0.59 |
| **C20:2 n-6** | 0 | 0.79 |
| **C20:3 n-6** | 0 | 0.12 |
| **C20:4 n-6** | 0 | 0.28 |
| **C20:5 n-3** | 0 | 0 |
| **C22:5 n-3** | 0 | 0.08 |
| **C22:6 n-3** | 0 | 0 |
| **SFA** | 18.2 | 32 |
| **MUFA** | 28.4 | 36 |
| **PUFA** | 53.4 | 32 |
| **n-6 PUFA** | 52.3 | 29.9 |
| **n-3 PUFA** | 1.1 | 2.1 |
| **n-6/n-3** | 47.6 | 14.1 |

SFA, Saturated fatty acids; MUFA, Monounsaturated fatty acids; PUFA, Polyunsaturated fatty acids

**Table S2.** Tail fatty acid profiles of mothers and offspring before and after high fat diet feeding

| **Tail fatty acid profile** | | | | | | | | | | |
| --- | --- | --- | --- | --- | --- | --- | --- | --- | --- | --- |
|  | **Mothers** | | **Pre-HFD (week 4)** | | | | **Post-HFD (week 17)** | | | |
|  | **WT** | ***fat-1*** | ***fat-1*/WT** | **WT/ *fat-1*** | ***fat-1 fat-1*** | **WT/WT** | ***fat-1*/WT** | **WT/ *fat-1*** | ***fat-1 fat-1*** | **WT/WT** |
| *SFA* |  |  |  |  |  |  |  |  |  |  |
| **12:0** | 0.6 ± 0.1 | 2.1 ± 0.3 | 3 ± 0.2 | 3 ± 0.2 | 2.3 ± 0.1 | 3 ± 0.2 | 1.4 ± 0^a^ | 1.2 ± 0^b^ | 1.3 ± 0^a^ ^b^ | 1.3 ± 0^a^ ^b^ |
| **14:0** | 0.1 ± 0 | 0.4 ± 0.1 | 0.2 ± 0 | 0.2 ± 0 | 0.1 ± 0 | 0.2 ± 0 | 0.3 ± 0 | 0.3 ± 0 | 0.3 ± 0 | 0.3 ± 0 |
| **16:0** | 14.8 ± 0.3^a^ | 18.3 ± 0.4^b^ | 16.4 ± 0.2 | 16.9 ± 0.2 | 16.2 ± 0.1 | 16.4 ± 0.3 | 12.7 ± 0.1 | 13 ± 0.2 | 12.7 ± 0.2 | 13 ± 0.4 |
| **17:0** | 0.5 ± 0 | 0.5 ± 0 | 0.5 ± 0 | 0.5 ± 0 | 0.5 ± 0 | 0.5 ± 0 | 0.2 ± 0^a^ | 0.2 ± 0^a^ ^b^ | 0.2 ± 0^a^ ^b^ | 0.2 ± 0^b^ |
| **18:0** | 10.2 ± 0.3 | 11.6 ± 0.4 | 10.8 ± 0.3 | 10.9 ± 0.4 | 10.8 ± 0.4 | 10.4 ± 0.3 | 3.4 ± 0.1^a^ | 4.4 ± 0.3^a^ ^b^ | 5 ± 0.7^b^ | 5 ± 0.4^b^ |
| **20:0** | 1.5 ± 0.1 | 1 ± 0.1 | 1.1 ± 0.1 | 0.9 ± 0 | 1 ± 0.1 | 1.1 ± 0.1 | 0.4 ± 0^a^ | 0.6 ± 0^a^ ^b^ | 0.5 ± 0^a^ | 0.6 ± 0.1^b^ |
| **22:0** | 2.5 ± 0.2 | 1.6 ± 0.1 | 2.4 ± 0.4 | 1.9 ± 0.1 | 2.4 ± 0.2 | 1.9 ± 0.1 | 0.6 ± 0^a^ | 0.6 ± 0^a^ | 0.5 ± 0^a^ | 0.9 ± 0.1^b^ |
| **24:0** | 3.1 ± 0.2 | 2.8 ± 0.2 | 3.7 ± 0.2 | 3.4 ± 0.2 | 3.5 ± 0.2 | 3.6 ± 0.2 | 1.1 ± 0.1^a^ ^c^ | 0.9 ± 0.1^b^ | 0.8 ± 0.1^b^ | 1.2 ± 0.1^c^ |
| **Total SFA** | 29.8 ± 0.5 | 38.2 ± 0.8 | 39.4 ± 0.4 | 39 ± 0.6 | 38.2 ± 1.2 | 38.3 ± 0.7 | 20.4 ± 0.2^a^ | 21.3 ± 0.4^a^ ^b^ | 21.4 ± 0.7^a^ ^b^ | 22.7 ± 0.7^b^ |
| *MUFA* |  |  |  |  |  |  |  |  |  |  |
| **14:1** | 0.4 ± 0 | 0.4 ± 0 | 0.4 ± 0 | 0.4 ± 0 | 0.4 ± 0 | 0.4 ± 0 | 0.2 ± 0^a^ | 0.2 ± 0^a^ | 0.2 ± 0^a^ ^b^ | 0.2 ± 0^b^ |
| **16:1** | 6 ± 0.7 | 5.4 ± 0.2 | 3.4 ± 0.1 | 3.6 ± 0.2 | 3.3 ± 0.2 | 3.5 ± 0.2 | 11.4 ± 0.3^a^ | 10.2 ± 0.3^b^ | 11.2 ± 0.2^a^ ^b^ | 10.4 ± 0.3^a^ ^b^ |
| **17:1** | 0.5 ± 0.1 | 0 ± 0 | 0.4 ± 0 | 0.4 ± 0 | 0.5 ± 0 | 0.4 ± 0 | 0.5 ± 0 | 0.5 ± 0 | 0.5 ± 0 | 0.5 ± 0 |
| **18:1** | 31.2 ± 1.4^a^ | 30.1 ± 0.3^b^ | 22.1 ± 0.4 | 22.7 ± 0.4 | 22.7 ± 0.5 | 22.8 ± 0.4 | 38.5 ± 0.3^a^ ^b^ | 39.3 ± 0.7^a^ | 37.9 ± 1.5^a^ ^b^ | 35.7 ± 0.4^b^ |
| **20:1** | 2.2 ± 0.1 | 2 ± 0.1 | 1.8 ± 0.1 | 1.7 ± 0.1 | 1.8 ± 0.1 | 2 ± 0.1 | 0.8 ± 0.1 | 1.3 ± 0.2 | 1.3 ± 0.1 | 1.3 ± 0.1 |
| **22:1** | 1.6 ± 0.2 | 1.5 ± 0.1 | 1.2 ± 0.1 | 1 ± 0.1 | 1.5 ± 0.3 | 1.3 ± 0.1 | 0.3 ± 0^a^ | 0.4 ± 0.1^a^ ^b^ | 0.5 ± 0.1^a^ ^b^ | 0.6 ± 0^b^ |
| **24:1** | 1.6 ± 0^a^ | 1.6 ± 0.1^b^ | 1 ± 0.1 | 1 ± 0.1 | 1.3 ± 0.1 | 1.1 ± 0 | 0.3 ± 0^a^ | 0.4 ± 0.1^a^ ^b^ | 0.3 ± 0^a^ ^b^ | 0.6 ± 0.1^b^ |
| **MUFA** | 43.4 ± 1.9^a^ | 40.9 ± 0.7^b^ | 30.3 ± 0.7 | 30.8 ± 0.7 | 31.4 ± 0.8 | 31.6 ± 0.5 | 52.1 ± 0.3^a^ ^b^ | 52.4 ± 0.8^a^ | 51.9 ± 1.3^a^ ^b^ | 49.3 ± 0.5^b^ |
| *PUFA* |  |  |  |  |  |  |  |  |  |  |
| *n-6 PUFA* |  |  |  |  |  |  |  |  |  |  |
| **18:2 n-6** | 15.5 ± 1 | 12 ± 0.6 | 16.5 ± 0.4 | 16.9 ± 0.4 | 16.9 ± 0.7 | 17.4 ± 0.3 | 20.9 ± 0.3 | 19.6 ± 0.4 | 20.5 ± 0.4 | 20 ± 0.3 |
| **18:3 n-6** | 0 ± 0^a^ | 0.2 ± 0^b^ | 0.1 ± 0 | 0.1 ± 0 | 0.1 ± 0 | 0.1 ± 0 | 0.1 ± 0 | 0.1 ± 0 | 0.1 ± 0 | 0.1 ± 0 |
| **20:2 n-6** | 1.2 ± 0.2 | 0.9 ± 0.1 | 1.2 ± 0.1 | 1.2 ± 0 | 1 ± 0 | 1.1 ± 0.1 | 0.7 ± 0 | 0.7 ± 0.1 | 0.8 ± 0 | 0.9 ± 0.1 |
| **20:3 n-6** | 0.3 ± 0.1 | 0.2 ± 0 | 0.7 ± 0 | 0.7 ± 0 | 0.5 ± 0.1 | 0.6 ± 0 | 0.3 ± 0 | 0.3 ± 0 | 0.3 ± 0 | 0.4 ± 0 |
| **20:4 n-6** | 4.7 ± 0.7^a^ | 0.8 ± 0.1^b^ | 9.1 ± 0.4 | 8.3 ± 0.4 | 8.8 ± 0.6 | 8.2 ± 0.4 | 2.7 ± 0.1^a^ | 2.6 ± 0.2^a^ | 2.3 ± 0.2^a^ | 3.4 ± 0.1^b^ |
| **22:4 n-6** | 0.7 ± 0.1 | 0 ± 0 | 1.7 ± 0.1 | 1.4 ± 0.1 | 1.4 ± 0.1 | 1.7 ± 0.1 | 0.5 ± 0^a^ ^b^ | 0.5 ± 0.1^a^ ^b^ | 0.5 ± 0^a^ | 0.7 ± 0^b^ |
| **Total n-6 PUFA** | 0.9 ± 0.2^a^ | 14 ± 0.6^b^ | 29.2 ± 0.9 | 28.6 ± 0.9 | 28.7 ± 1.5 | 29.2 ± 0.9 | 25.3 ± 0.3 | 23.8 ± 0.6 | 24.4 ± 0.6 | 25.4 ± 0.3 |
| *n-3 PUFA* |  |  |  |  |  |  |  |  |  |  |
| **18:3 n-3** | 0.1 ± 0 | 2.2 ± 0.2 | 0.1 ± 0 | 0.1 ± 0 | 0.1 ± 0 | 0.1 ± 0 | 1.2 ± 0^a^ ^b^ | 1 ± 0^b^ | 1.3 ± 0.1^a^ | 1.3 ± 0^a^ |
| **20:3 n-3** | 0 ± 0^a^ | 0.2 ± 0^b^ | 0 ± 0 | 0 ± 0 | 0 ± 0 | 0 ± 0 | 0.1 ± 0 | 0.1 ± 0 | 0.1 ± 0 | 0.2 ± 0 |
| **20:5 n-3** | 0 ± 0^a^ | 1.7 ± 0.2^b^ | 0 ± 0 | 0 ± 0 | 0 ± 0 | 0 ± 0 | 0.1 ± 0 | 1.2 ± 0.7 | 0 ± 0 | 0.1 ± 0 |
| **22:5 n-3** | 0 ± 0^a^ | 1.7 ± 0.2^b^ | 0.2 ± 0^a^ | 0.4 ± 0.1^b^ | 0.4 ± 0.1^b^ | 0.2 ± 0^a^ | 0.3 ± 0^a^ | 0.2 ± 0^a^ ^b^ | 0.2 ± 0^b^ | 0.3 ± 0^a^ ^b^ |
| **22:6 n-3** | 0.8 ± 0.2 | 1.3 ± 0.1 | 0.9 ± 0^a^ ^b^ | 1.1 ± 0.1^b^ ^c^ | 1.2 ± 0.1^c^ | 0.7 ± 0.1^a^ | 0.7 ± 0.1 | 0.8 ± 0.1 | 0.7 ± 0.1 | 0.8 ± 0.1 |
| **Total n-3 PUFA** | 22.5 ± 1.8^a^ | 7 ± 0.6^b^ | 1.1 ± 0.1 | 1.6 ± 0.2 | 1.7 ± 0.1 | 0.9 ± 0.1 | 2.3 ± 0.1 | 2.5 ± 0.3 | 2.3 ± 0.1 | 2.5 ± 0.1 |
| **n-6/n-3** | 38.1 ± 8^a^ | 2.1 ± 0.2^b^ | 26.7 ± 1.1 | 18.6 ± 1.5 | 16.8 ± 1 | 33.5 ± 2.6 | 11.3 ± 0.3 | 10.3 ± 0.8 | 10.7 ± 0.4 | 10 ± 0.3 |

SFA, Saturated fatty acids; MUFA, Monounsaturated fatty acids; PUFA, Polyunsaturated fatty acids; HFD, High fat diet. Values with different superscript letters are significantly different between groups within timepoint. Data expressed as mean ± SEM. n=5-10 per grou

**Table S3.** Liver fatty acid profiles of offspring after high fat diet feeding.

|  | **Liver** | | | |
| --- | --- | --- | --- | --- |
|  | ***fat-1*/WT** | **WT/*fat-1*** | ***fat-1*/*fat-1*** | **WT/WT** |
| *SFA* |  |  |  |  |
| **12:0** | 0.5 ± 0 | 0.5 ± 0 | 0.5 ± 0 | 0.5 ± 0 |
| **14:0** | 0 ± 0 | 0 ± 0 | 0 ± 0 | 0 ± 0 |
| **16:0** | 26.8 ± 0.3 | 25.9 ± 0.6 | 26.8 ± 0.8 | 25.1 ± 0.8 |
| **17:0** | 0.2 ± 0 | 0.2 ± 0 | 0.2 ± 0 | 0.2 ± 0 |
| **18:0** | 3.7 ± 0.2 | 5.5 ± 0.6 | 5 ± 0.5 | 4 ± 0.4 |
| **20:0** | 0.1 ± 0^a^ | 0.2 ± 0^b^ | 0.2 ± 0^b^ ^c^ | 0.2 ± 0^a^ ^c^ |
| **22:0** | 0.1 ± 0 | 0.2 ± 0 | 0.2 ± 0 | 0.1 ± 0 |
| **24:0** | 0 ± 0 | 0 ± 0 | 0 ± 0 | 0 ± 0 |
| **Total SFA** | 31.4 ± 0.4 | 32.6 ± 0.8 | 32.9 ± 1.3 | 30 ± 1.1 |
| *MUFA* |  |  |  |  |
| **14:1** | 0.1 ± 0 | 0.1 ± 0 | 0.1 ± 0 | 0.1 ± 0 |
| **16:1** | 3.2 ± 0.3 | 2.6 ± 0.2 | 2.6 ± 0.2 | 2.9 ± 0.2 |
| **17:1** | 0.3 ± 0 | 0.3 ± 0 | 0.3 ± 0 | 0.3 ± 0 |
| **18:1** | 42.2 ± 0.6 | 36.5 ± 1.6 | 38.2 ± 1.9 | 39.3 ± 1.6 |
| **20:1** | 1 ± 0.1 | 0.9 ± 0 | 0.9 ± 0.1 | 1 ± 0.1 |
| **22:1** | 0.1 ± 0 | 0.1 ± 0 | 0.1 ± 0 | 0.1 ± 0 |
| **24:1** | 0 ± 0 | 0 ± 0 | 0 ± 0 | 0 ± 0 |
| **MUFA** | 46.8 ± 0.9 | 40.4 ± 1.8 | 42 ± 2.2 | 43.6 ± 1.8 |
| *PUFA* |  |  |  |  |
| *n-6 PUFA* |  |  |  |  |
| **18:2 n-6** | 15.3 ± 0.4^a^ | 17.7 ± 0.6^a^ ^b^ | 17.4 ± 0.7^a^ ^b^ | 18 ± 0.5^b^ |
| **18:3 n-6** | 0.3 ± 0 | 0.3 ± 0 | 0.4 ± 0 | 0.4 ± 0 |
| **20:2 n-6** | 0.3 ± 0 | 0.3 ± 0 | 0.3 ± 0 | 0.4 ± 0 |
| **20:3 n-6** | 0.8 ± 0^a^ | 0.9 ± 0^a^ ^b^ | 0.9 ± 0.1^a^ ^b^ | 1 ± 0^b^ |
| **20:4 n-6** | 2.7 ± 0.1 | 4.4 ± 0.7 | 3.5 ± 0.3 | 3.5 ± 0.3 |
| **22:4 n-6** | 0.3 ± 0 | 0.3 ± 0 | 0.3 ± 0 | 0.4 ± 0 |
| **Total n-6 PUFA** | 19.7 ± 0.7^a^ | 24.1 ± 1.3^b^ | 22.7 ± 1.1^a^ ^b^ | 23.6 ± 0.7^a^ ^b^ |
| *n-3 PUFA* |  |  |  |  |
| **18:3 n-3** | 0.4 ± 0 | 0.5 ± 0 | 0.5 ± 0 | 0.5 ± 0 |
| **20:3 n-3** | 0.1 ± 0 | 0 ± 0 | 0 ± 0 | 0 ± 0 |
| **20:5 n-3** | 0.1 ± 0 | 0.2 ± 0 | 0.1 ± 0 | 0.1 ± 0 |
| **22:5 n-3** | 0.3 ± 0 | 0.3 ± 0 | 0.3 ± 0 | 0.3 ± 0 |
| **22:6 n-3** | 1.3 ± 0.1 | 2 ± 0.4 | 1.6 ± 0.2 | 1.8 ± 0.1 |
| **Total n-3 PUFA** | 2.1 ± 0.2 | 3 ± 0.5 | 2.4 ± 0.3 | 2.8 ± 0.2 |
| **n-6/n-3** | 9.7 ± 0.7 | 9.1 ± 0.8 | 10.1 ± 0.8 | 8.7 ± 0.4 |

SFA, Saturated fatty acids; MUFA, Monounsaturated fatty acids; PUFA, Polyunsaturated fatty acids. Values with different superscript letters are significantly different between groups. Data expressed as mean ± SEM. n=5-10 per group

**Table S4.** Primer sequences for qPCR

| **Primers for qPCR** | | |
| --- | --- | --- |
| **Gene** | **Forward** | **Reverse** |
| *TNFa* | TGGGACAGTGACCTGGACTGT | TTCGGAAAGCCCATTTGAGT |
| *F4/80* | TGACAACCAGACGGCTTGTG | GCAGGCGAGGAAAAG ATAGTGT |
| *CCL2* | AGGTCCCTGTCATGCTTCTGG | CTGCTGCTGGTGATCCTCTTG |
| B-actin | GACCCAGATCATGTTTGAGA | GAGCATAGCCCTCGTAGAT |
| *TLR4* | ACCAGGAAGCTTGAATCCCT | TCCAGCCACTGAAGTTCTGA |

**SUPPLEMENTARY METHODS**

**Glucose tolerance test**

Glucose tolerance test (GTT) was performed as previously described (Kaliannan et al., 2013) at weaning (PND 28) and following 13-15 weeks on a HFD. Due to the age and size of the animals at weaning, a number of small modifications were made to the GTT procedure compared with that performed at adulthood. Briefly, fasting blood glucose was measured using a glucometer following 5-6 h fasting. Glucose [20% solution in ddH_2_0 (weaning); 10% solution in phosphate buffered saline (post-HFD)] was administered at 1g/kg body weight (weaning) or 2g/kg body weight (post-HFD) by i.p. injection (weaning) or gavage (post-HFD). A small incision was made in the tail and blood samples were collected at 15, 30, 60, 90 and 120 min post glucose administration to measure glucose levels. Glucose tolerance was assessed by calculating the incremental area under the curve (AUC) of the GTT.

**Insulin tolerance test**

Insulin tolerance test (ITT) was performed as previously described (Kaliannan et al., 2013) following 13-15 weeks on a HFD. Briefly, fasting blood glucose was measured using a glucometer following 6 h fasting. Insulin (solution in PBS) was administered at 0.75 units/kg body weight by i.p. injection. A small incision was made in the tail and blood samples were collected at 10, 20, 30, 40, 50 and 60 min post insulin administration to measure glucose levels. Insulin tolerance was assessed by calculating the incremental AUC of the ITT. The homeostasis model assessment of insulin resistance [HOMA-IR = glucose (mg/dL) × insulin (mU/L)/405] was calculated as an insulin sensitivity index.

**Cytokines**

Serum cytokine levels (TNF-α, IL-1β, IL-6, MCP-1, and IL-10) were assessed using a Bio-Plex immunoassay kit (Bio-Rad) according to manufacturer’s instructions. Data acquisition and analysis was performed using Xponent softward (Luminex, Austin, TX).

**Similarity Percentage (SIMPER) analysis**

Taxa which are primarily responsible for an observed difference between groups were identified by Similarity Percentage (SIMPER) analysis method and their contribution to groups (between and within groups) were analyzed using Principal Component (PCA) variance-covariance type ordination (PAST v3.18) method.

**qPCR**

RNA extraction from tissue samples was carried out using TRIzol reagent (Invitrogen Life Technologies, Grand Island, NY), following the manufacturer’s instructions. RNA concentration (ng/ul) and purity (A260/280) of each sample was assessed using a plate reader. cDNA synthesis of RNA was performed using the iScript cDNA Synthesis Kit (Bio-Rad) according to manufacturers instructions. qPCR was performed using SYBR Green reagent in a PRISM 9000 Light Cycler (Applied Biosystems). Primer sequences are detailed in Table S7. qPCR reactions were performed in triplicate and normalized to a house-keeping gene (β-actin). Gene expression was calculated using the ΔΔCt method.
